# Full Inhibition of Spinal FAAH Leads to TRPV1-Mediated Analgesic Effects in Neuropathic Rats and Possible Lipoxygenase-Mediated Remodeling of Anandamide Metabolism

**DOI:** 10.1371/journal.pone.0060040

**Published:** 2013-04-03

**Authors:** Katarzyna Starowicz, Wioletta Makuch, Michal Korostynski, Natalia Malek, Michal Slezak, Magdalena Zychowska, Stefania Petrosino, Luciano De Petrocellis, Luigia Cristino, Barbara Przewlocka, Vincenzo Di Marzo

**Affiliations:** 1 Dept. of Pain Pharmacology, Institute of Pharmacology, Polish Academy of Sciences, Smetna, Krakow, Poland; 2 Dept. of Molecular Neuropharmacology, Institute of Pharmacology, Polish Academy of Sciences, Smetna, Krakow, Poland; 3 Endocannabinoid Research Group, Istituto di Chimica Biomolecolare, CNR, Pozzuoli (Naples), Italy; 4 Endocannabinoid Research Group, Istituto di Cibernetica, CNR, Pozzuoli (Naples), Italy; Centre for Addiction and Mental Health, Canada

## Abstract

Neuropathic pain elevates spinal anandamide (AEA) levels in a way further increased when URB597, an inhibitor of AEA hydrolysis by fatty acid amide hydrolase (FAAH), is injected intrathecally. Spinal AEA reduces neuropathic pain by acting at both cannabinoid CB1 receptors and transient receptor potential vanilloid-1 (TRPV1) channels. Yet, intrathecal URB597 is only partially effective at counteracting neuropathic pain. We investigated the effect of high doses of intrathecal URB597 on allodynia and hyperalgesia in rats with chronic constriction injury (CCI) of the sciatic nerve. Among those tested, the 200 µg/rat dose of URB597 was the only one that elevated the levels of the FAAH non-endocannabinoid and anti-inflammatory substrates, oleoylethanolamide (OEA) and palmitoylethanolamide (PEA), and of the endocannabinoid FAAH substrate, 2-arachidonoylglycerol, and fully inhibited thermal and tactile nociception, although in a manner blocked almost uniquely by TRPV1 antagonism. Surprisingly, this dose of URB597 decreased spinal AEA levels. RT-qPCR and western blot analyses demonstrated altered spinal expression of lipoxygenases (LOX), and baicalein, an inhibitor of 12/15-LOX, significantly reduced URB597 analgesic effects, suggesting the occurrence of alternative pathways of AEA metabolism. Using immunofluorescence techniques, FAAH, 15-LOX and TRPV1 were found to co-localize in dorsal spinal horn neurons of CCI rats. Finally, 15-hydroxy-AEA, a 15-LOX derivative of AEA, potently and efficaciously activated the rat recombinant TRPV1 channel. We suggest that intrathecally injected URB597 at full analgesic efficacy unmasks a secondary route of AEA metabolism via 15-LOX with possible formation of 15-hydroxy-AEA, which, together with OEA and PEA, may contribute at producing TRPV1-mediated analgesia in CCI rats.

## Introduction

The endocannabinoid system includes the cannabinoid CB_1_ and CB_2_ receptors (two G-protein-coupled receptors activated also by the main psychotropic component of *Cannabis sativa*, Δ^9^-tetrahydrocannabinol [1]), and the endogenous agonists at these receptors, i.e. the endocannabinoids anandamide (AEA) and 2-arachidonoyl glycerol (2-AG)]. AEA also activates the transient receptor potential vanilloid-1 (TRPV1) channel [2], which transduces the pronociceptive and heat-like effects of another plant natural product, the pungent component of hot peppers, capsaicin [3], [4], [5], [6]. TRPV1 usually, but not always, plays in pain transmission a role opposite to cannabinoid receptors [7], [8] and, therefore, the possible effects on pain of the activation of this channel by AEA have been thoroughly studied [9], [10], [11].

Enzymes for endocannabinoid catabolism have been characterized. Fatty acid amide hydrolase (FAAH) catalyzes the hydrolysis of AEA and its two congeners, the acylethanolamides oleoylethanolamide (OEA) and palmitoylethanolamide (PEA), which occur in higher tissue concentrations than AEA and produce their effects in a mostly cannabinoid receptor-independent manner. FAAH catalyzes also the hydrolysis of 2-AG [12], [13], which, however, is usually degraded in vivo by monoacylglycerol lipase [14]. Additionally, lipoxygenase (LOX)- and cyclooxygenase (COX)-2- catalyzed oxygenation of endocannabinoids generate several bioactive compounds [15], [16]. Whilst COX-2 derivatives are inactive at cannabinoid receptors and TRPV1 [16], LOX products of AEA are still able to activate these targets [17], [18].

TRPV1 and CB_1_ co-localization in the spinal cord and dorsal root ganglia opens interesting possibilities for the development of novel therapeutics against neuropathic pain. Administration of FAAH inhibitors, like URB597 [19], to rodents enhances the levels of AEA, PEA, OEA and/or 2-AG in various tissues involved in pain perception [20], [21], [22]. Depending on the dose of URB597, or the time after its injection in the periaqueductal grey, either a suppression or an increase in thermal nociception via TRPV1 or CB_1_ receptors, respectively, was observed [21]. Intrathecal injection of URB597 elevates AEA levels and produces antihyperalgesic and anti-allodynic effects in neuropathic rats via CB_1_ or TRPV1-mediated mechanisms depending on the dose used [23]. Therefore, local injection of FAAH inhibitors offers the opportunity to investigate the role of FAAH substrates and their molecular targets in pain control. Yet, the possibility that full FAAH inhibition unmasks other pathways for AEA metabolism, with likely consequences on nociception, has been poorly investigated (see [24] for a rare example). Therefore, the present study aimed at elucidating the mechanism of action of a fully effective analgesic dose of URB597 in rats with chronic constriction injury (CCI) of the sciatic nerve, by establishing how it modifies endocannabinoid and acylethanolamide levels in the spinal cord and determining the contribution of CB_1_, TRPV1 and LOX to its effects.

## Results

### Anti-allodynic and Anti-hyperalgesic Effects of Intrathecal Administration of the FAAH Inhibitor, URB597 in CCI Rats

**Figure 1:**
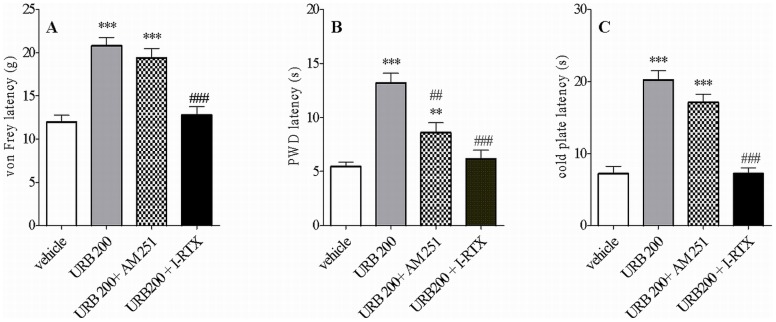
Effect of URB597 (a selective inhibitor of the enzyme fatty acid amide hydrolase, FAAH) at the dose of 200 µg, on mechanical allodynia (A), thermal hyperalgesia (B) and cold allodynia (C) in CCI rats. Experiments were conducted 7 days after the CCI. Antagonists (I-RTX or AM251) were administered 15 min before URB597 and behavioral evaluations were made 30 min after the last administration. The doses of I-RTX and AM251 used for antagonism of URB597 effects had no effect on allodynia and hyperalgesia *per se* (for details, please refer to [23]). All drugs were given i.t. in a volume of 10 µl. Data represents mean values ± SEM (n = 8). Statistical analyses were performed with one-way ANOVA with Bonferroni as post test. **p<0.01; ***p<0.001 versus vehicle-treated rats (veh); ##p<0.01 and ###p<0.001 versus URB597-treated rats.

We assessed the selective FAAH inhibitor, URB597, on signs of neuropathic pain in rats subjected to CCI. URB597 at doses of 10 and 100 µg was effective at reducing mechanical allodynia (p<0.001) and thermal hyperalgesia (p<0.001) as well as cold allodynia (p<0.001) ([23] and data not shown). However, URB597 was maximally effective at exerting these effects at the dose of 200 µg, when monitoring mechanical allodynia (Fig. 1A, p<0.001, 174% over vehicle), thermal hyperalgesia (Fig. 1B, p<0.001, 242% over vehicle) and cold allodynia (Fig. 1C, p<0.001, 281% over vehicle) (Fig. 1). The analgesic effects observed with this high dose of URB597 were significantly attenuated by pretreatment with the selective TRPV1 antagonist, 5'-iodoresiniferatoxin (I-RTX), starting at 15 min after i.t. injection and still present at 60 min (data not shown). I-RTX significantly reduced URB597-mediated tactile allodynia, thermal hyperalgesia and cold allodynia. Pretreatment with the CB_1_ receptor antagonist, AM251, affected URB597 (200 µg)–mediated analgesia in Hargreaves test only at 30 min after i.t. injection (Fig. 1B). All remaining values from rats pretreated with AM251 did not differ significantly from those treated with URB597 (200 µg) alone (Fig. 1 A–C).

Both types of treatment (URB597 200 µg alone or preceeded by I-RTX or AM251) and time after injection yielded statistically different effects. Data analysis were as follows: for tactile allodynia (drug injected: F_3,112_ = 29.67; time: F_3,112_ = 13.36; parameters mutually *p*<0.001); for Hargreaves test (drug: F_3,112_ = 17.29; time: F_3,112_ = 7.05; parameters *p*<0.0001 and *p*<0.0002, respectively); for cold plate test (drug: F_3,112_ = 29.27; time: F_3,112_ = 13.33; parameters mutually *p*<0.0001). Neither I-RTX nor AM251 had effect on allodynia *per se* at the doses used (for details see also: [23]).

### Effect of FAAH Inhibition on the Levels of Endogenous AEA, Related Fatty Acid Amides and 2-AG in the Spinal Cord of CCI Rats

**Figure 2:**
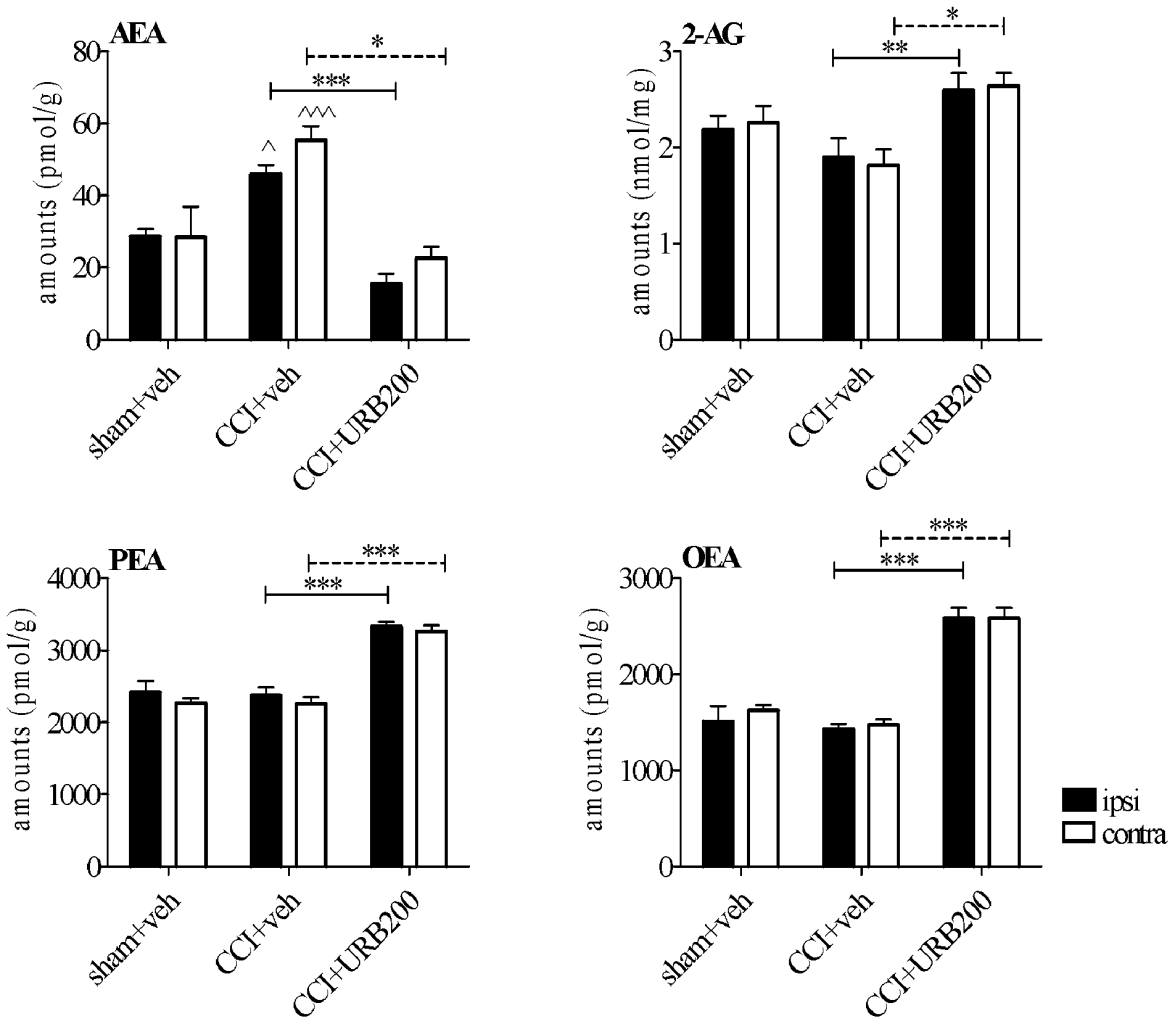
LC-MS analysis of anandamide (AEA),2*-*arachidonoylglycerol(2-AG), palmitoylethanolamide (PEA) and oleoylethanolamide (OEA) in the ipsilateral (black bars) and contralateral (open bars) parts of the lumbar spinal cords of sham-operated and CCI rats in response to pharmacological modulation of its endogenous levels evoked by URB597 (200 µg) administration. Experiments were conducted 7 days after the CCI.URB597 was given i.t. in a volume of 10 µl. Data are means ± SEM of n = 6 rats. Means were compared by two-way ANOVA with side and treatment as a variable factors with Bonferroni as post test. ∧*p*<0.05; ∧∧∧*p*<0.001 versus sham-operated, vehicle (veh) injected group, *p<0.05; **p<0.01 and ***p<0.001 versus CCI, veh injected rats.

In separate experiments, we assessed the levels of AEA, 2-AG, PEA and OEA in the lumbar spinal cord of sham-operated and CCI rats treated with URB597 vs. vehicle (Fig. 2). Seven days after CCI, AEA levels were increased both in the ipsi- and contralateral side (Fig. 1A, 60.6% and 95% elevation *vs*. sham-operated rats, *p*<0.05 and *p*<0.001, respectively). By contrast, administration of URB597 at the dose of 200 µg resulted in a significant decrease in spinal AEA levels (ipsilateral: 45.5% reduction vs. sham-vehicle rats, *p*<0.05 and 66% reduction vs. CCI-vehicle rats, *p*<0.001; contralateral: 20% reduction vs. sham-vehicle rats, p>0.05 and 59% reduction vs. CCI- vehicle rats, *p*<0.05).

Intrathecal delivery of URB597 (200 µg) resulted in a significant increase of 2-AG levels, both ipsi- and contralateral (36.8% (*p*<0.05) and 45.5% (*p*<0.01)) *vs*. CCI-vehicle rats (Fig. 2B). Likewise, we observed a significant increase in PEA levels by 40% at the ipsilateral side (*p*<0.001) and 44% at the contralateral side (*p*<0.001) *vs*. CCI-vehicle rats (Fig. 2C). URB597 treatment (200 µg) caused a significant elevation also of OEA by 80% and 75% *vs*. CCI-vehicle rats (Fig. 2D, *p*<0.001 mutually).

Two-way analysis of variance resulted in the general conclusion that only the treatment procedure, but not side of the injury (ipsi- *vs*. contralateral), affected the presented results in a significant manner.

### Gene Expression Level of the Major Lipoxygenases in CCI Rats

**Figure 3:**
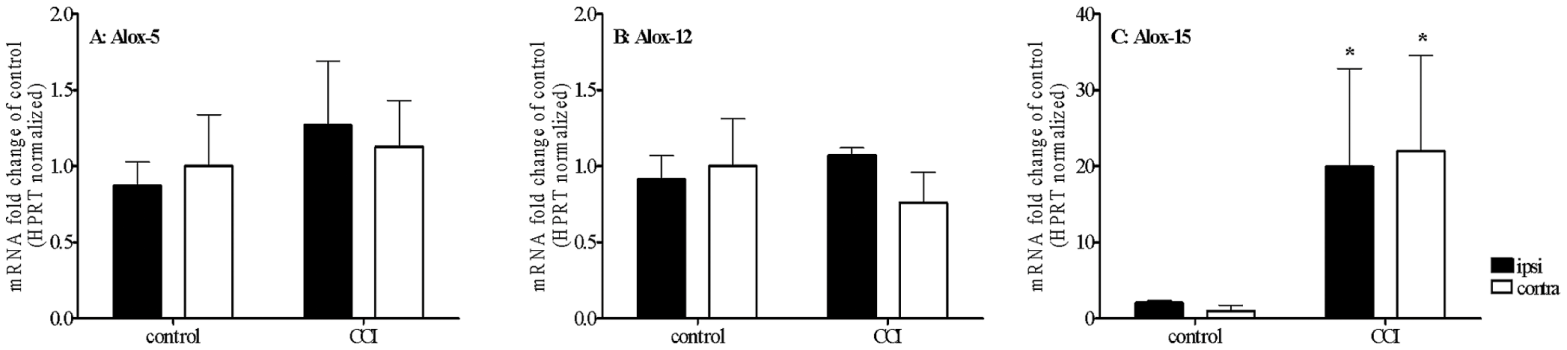
The results of qPCR analyses of LOX-15, LOX-12 and LOX-5 gene expression levels in the dorsal part of the lumbar (L5–L6) spinal cord of neuropathic rats. Tissue was dissected 7 days after the CCI. The data are presented as means251658240±251658240SEM, which represent normalized averages derived from 4–6 samples per each group. Statistical analysis was performed using a two-way ANOVA followed by Bonferroni post-test (*p<0.05).

Quantitative PCR (qPCR) analysis revealed that the abundance levels of *Alox15* (15-LOX) transcript were highly elevated (20-fold change versus control, *p*<0.05) seven days after CCI (Fig. 3C). The effects were observed in the lumbar spinal cord, both ipsi- and contralateral to the injury. The mRNA levels of *Alox5* (5-LOX, Fig. 3A) and *Alox12* (12-LOX, Fig. 3B) did not significantly change between the experimental groups.

### 15-LOX Protein Expression in the Rat Dorsal Horn

**Figure 4:**
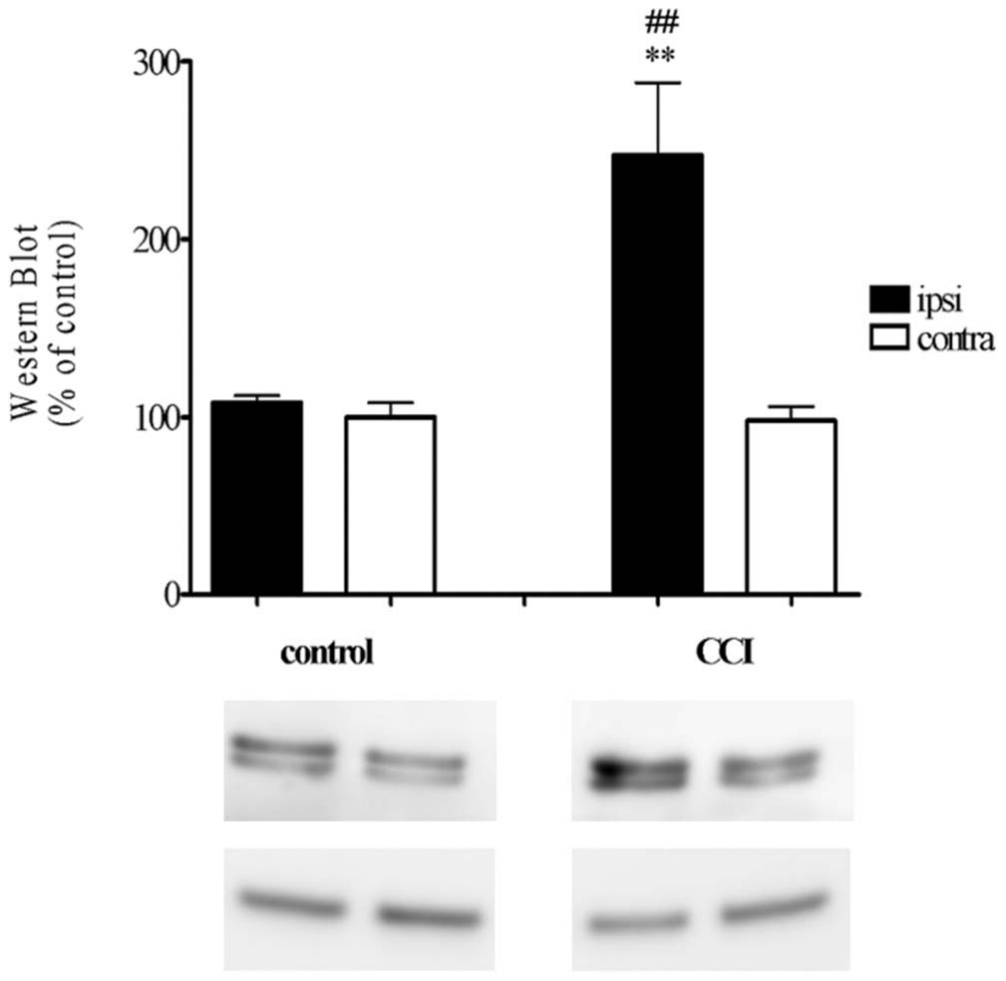
Western blot analysis of LOX-15 protein levels measured in the lumbar dorsal horn (L5–L6) of the spinal cord on day 7 of control (sham) and CCI in rats. The data are presented as means ± SEM, which represent GAPDH (lower line) normalized averages derived from 6 samples per each group Results are presented as a % of control densitometry analysis of all sections. Statistical analyses were performed with one-way ANOVA with Bonferroni as post-hoc test. **p<0.01 ipsilateral versus contralateral of CCI-exposed rats and ## p<0.01 CCI ipsilateral versus control (naïve and sham) rats.

To determine whether up-regulation of 15-LOX transcript in the lumbar dorsal horn (DH) was accompanied by corresponding changes in its protein levels, Western blot analysis was performed at 7 days after CCI, i.e. at the same time as mRNA measurements. In the control DH, 15-LOX proteins of 73 kDa were found. No significant differences from control animals were observed between ipsi and contralateral DH of naïve and sham-vehicle animals. A significant elevation of 15-LOX protein was observed exclusively on the ipsilateral side (249% vs. CCI contralateral; 247% vs. naïve and sham) (Fig. 4).

### Effect of Baicalein, an Inhibitor of LOX Activity, on the Analgesic Effects of URB597 in CCI Rats

**Figure 5:**
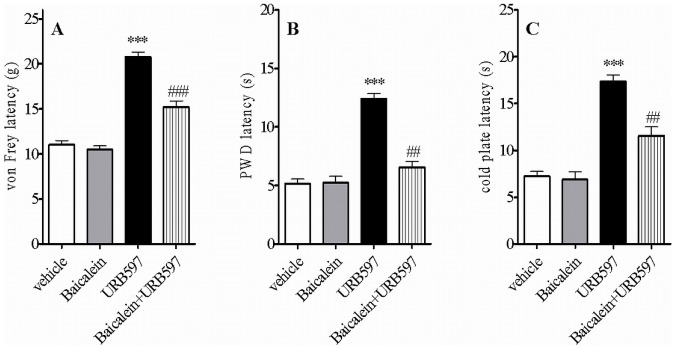
Effect of baicalein, an antioxidant 12/15-lipoxygenase inhibitor, on the behavioral effects of URB597 (200 µg) on mechanical allodynia (A), thermal hyperalgesia (B) and cold allodynia (C) in CCI rats. Experiments were conducted 7 days after the CCI. Baicalein was administered 15 min before URB597 and behavioral evaluations were made 30 min after the last administration. The dose of baicalein used for antagonism of URB597 had no effect on allodynia and hyperalgesia *per se*. Drugs were given i.t. in a volume of 10 µl. Data represents mean values ± SEM (n = 8). Statistical analyses were performed with one-way ANOVA with Bonferroni as post test. **p<0.01; ***p<0.001 versus vehicle-treated rats (veh); ##p<0.01 and ###p<0.001 versus URB597-treated rats.

The 12/15-LOX inhibitor, baicalein (1.5 µg), produced no effects in the absence of URB597 following CCI (Fig. 5A–C). Intrathecal injection of baicalein (1.5 µg) 15 min before URB597 (200 µg) injection highly and significantly reduced its anti-analgesic and anti allodynic actions (Fig. 5A–C, *p*<0.001 for von Frey *F*(_3,31_) 95.7, Hargreaves *F*(_3,31_) 54.3 and cold plate tests *F*(_3,31_) 40.5). However, all drug combinations were still active at attenuating neuropathic pain signs in rats (*p*<0.001 for von Frey test and *p*<0.01 for Hargreaves and cold plate tests).

### FAAH, TRPV1 and 15-LOX are Highly Colocalized in the Lumbar Spinal Cord of CCI Rats

**Figure 6:**
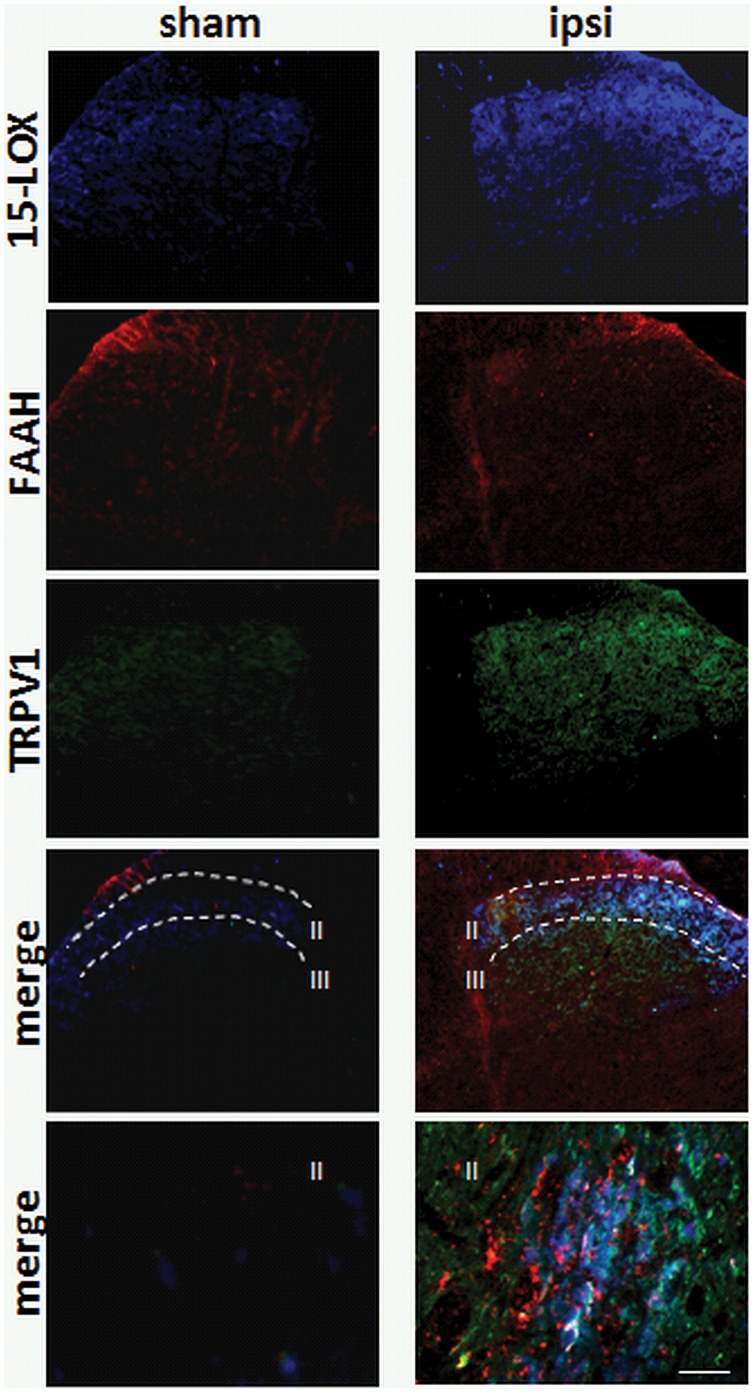
Effect of sciatic nerve CCI on 15-LOX, FAAH and TRPV1 immunoreactivities in sham operated and ipsilateral lumbar dorsal horn of the spinal cord. After sciatic nerve-CCI, a significant increase of 15-LOX/TRPV1 immunoexpression is observed especially in lamina II. FAAH immunoreactivity is located both in fibers and puncta in the cytoplasm of 15-LOX/TRPV1 neurons. Scale bar = 100 µm, and 30 µm in merged images.

Immunohistochemical localization of TRPV1 receptor, FAAH and, in view of the results of the q-PCR analyses, 15-LOX, was measured in the DH of CCI and sham operated rats by means of immunofluorescence (Fig. 6). In the lumbar DH lamina II/III, both in the sham and controlateral side to CCI spinal cord we observed relatively few TRPV1-positive (15.5±3.4%) or 15-LOX-positive (18.4±2.6%) neurons, whereas FAAH was more abundant, especially in the fibers and scattered puncta in the lamina II. Noteworthy, no significant double or triple immunoexpression was found in the controlateral CCI or sham lumbar spinal cord. In the ispilateral side to CCI we found a significant increase of both TRPV1 and 15-LOX immunoreactivity in the lumbar spinal cord. A density of 78.3±8.5% TRPV1-positive and 65.5±10.5% 15-LOX positive neurons were observed within lamina II/III of the DH, with main localization of 15-LOX neurons in lamina II. TRPV1 was co-localized with 15-LOX in 86.8±14.5% of the cell bodies of lamina II. Among these cell bodies, 50.5±8.7% showed 15-LOX/FAAH/TRPV1 co-localization (Fig. 6), thus demonstrating that TRPV1-ir neurons in the DH of rats with CCI might metabolize AEA via both 15-LOX and FAAH. FAAH staining was also observed in fibres (Fig. 6).

### The 15-LOX Derivative of AEA, 15(S)-hydroxy-AEA, Stimulates Intracellular Calcium Elevation via TRPV1

**Figure 7:**
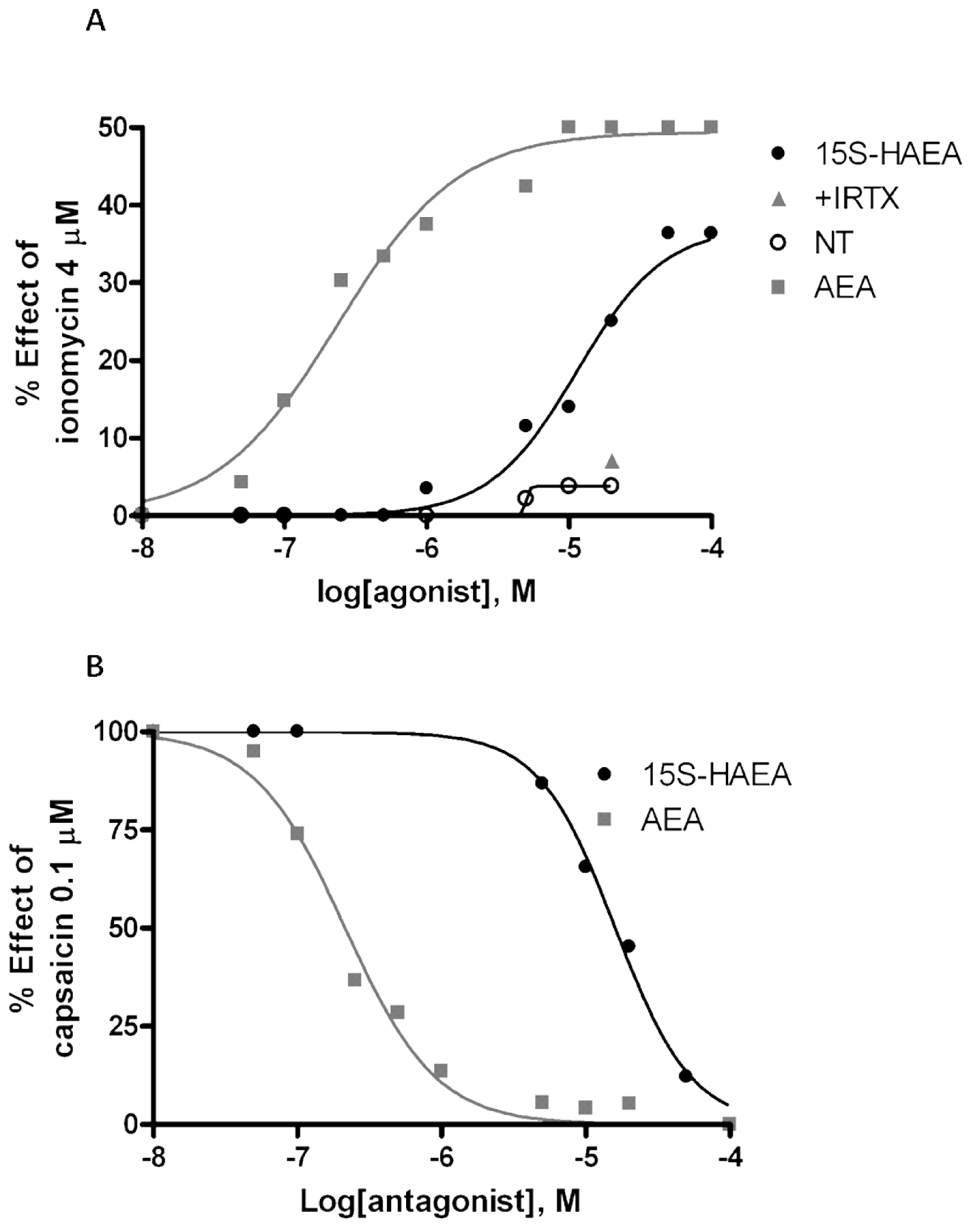
Effect of anandamide (squares) and 15(*S*)-hydroxy-anandamide (15S-HAEA) (filled circles) on intracellular Ca^2+^ elevation in HEK-293 cells overexpressing the rat recombinant TRPV1 channel (A). The effect of 5 min pre-incubation with the TRPV1 antagonist iodo-resiniferatoxin (I-RTX) (triangle), tested against 2 µM 15S-HAEA, and the effect of 15S-HAEA in wild-type HEK293 (i.e. not transfected with the TRPV1 construct, NT) are also shown. Desensitization, by 5 min pre-incubation with anandamide (squares) or15(*S*)-hydroxy-anandamide (15S-HAEA) (filled circles), of capsaicin (0.1 µM)-induced Ca^2+^ elevation in HEK-293 cells overexpressing the rat recombinant TRPV1 channel (B). In (A) data are expressed as % of the effect of ionomycin (4 µM, ‘maximal response’), and in (B) as % of the effect of capsaicin (0.1 µM). Data are means of n = 3 separate determinations. Standard error bars are not shown for the safe of clarity and were never higher than 10% of the means.

The addition of 15(*S*)-hydroxy-AEA activates to HEK-293 cells stably transfected with the rat recombinant TRPV1 caused intracellular Ca^2+^ elevation with an efficacy that was 37.3±0.8% of that of 4 µM ionomycin, and a potency (EC_50_) of 11.3±0.9 µM (Fig. 7A). Under the same conditions, AEA exhibited higher efficacy (49.5±0.9%) and potency (0.24±0.03 µM). To further characterise the specificity of TRPV1 agonism of 15(*S*)-hydroxy-AEA, we evaluated the effect on [Ca^2+^]_i_ either in wild type HEK293 (i.e. not transfected with the TRPV1 construct, WT) or after pre-treatment with the TRPV1 antagonist I-RTX [25]. In both cases, 15(*S*)-hydroxy-AEA exhibited much lower efficacy, if any. Finally, 15(*S*)-hydroxy-AEA was also able to desensitise TRPV1-mediated elevation of [Ca^2+^]_i_ induced by capsaicin (0.1 µM), with an IC_50_ = 16.0±0.5 µM. Again, AEA was more potent at desensitizing TRPV1 to the effect of capsaicin (Fig. 7B).

## Discussion

In the present study, the spinal administration of a high dose of the FAAH inhibitor, URB597, caused anti-allodynic and anti-hyperalgesic effects in neuropathic rats. The effects of URB597 were blocked by a selective TRPV1 receptor antagonist (I-RTX) and significantly attenuated by a 12/15-LOX inhibitor (baicalein). CCI was accompanied by increased levels of AEA and increased co-localization of TRPV1 with AEA catabolic enzymes: 15-LOX and FAAH in the lumbar spinal cord. The analgesic effects of URB597 (200 µg) were accompanied by increased spinal levels of PEA, OEA and 2-AG and, surprisingly, decreased levels of AEA as compared with matched vehicle-treated CCI controls. Our pharmacological and molecular data support the possibility that the full inactivation of FAAH, together with CCI-induced over-expression of TRPV1 and 15-LOX in the DH of the spinal cord, trigger: 1) an alternative pathway of AEA metabolism leading to15-LOX-derived metabolites that inhibit nociception via TRPV1 desensitization; and 2) elevation of the levels of AEA-related acylethanolamides with possibly similar TRPV1-mediated analgesic actions, i.e. PEA and OEA. Our findings, together with our previous investigation of the effects of lower intrathecal doses of URB597, or of varying doses of AEA [23], suggest that elevation of spinal AEA levels with increasing doses of a locally injected FAAH inhibitor, produce anti-hyperalgesic and anti-allodynic actions via mechanisms that progressively involve TRPV1 channel desensitization rather than CB_1_ receptor activation (CB_2_ receptors are not a likely target for cannabinoid-mediated antinociception in CCI model and AEA is only a partial agonist at CB2 receptors [26], [27], [28]). FAAH inhibition would result first in elevation of AEA levels to an extent that is high enough to activate/desensitize this channel [23], and then in the elevation of the levels of other TRPV1-active acylethanolamides and in the activation of AEA catabolic pathways leading to other TRPV1-active metabolites (present data). Thus, potent inhibition of FAAH may cause fully effective analgesic actions against neuropathic pain as a result of a complex readaptation of endocannabinoid metabolism and signaling, involving AEA-lipoxygenation, PEA and OEA.

It should be emphasized that the concentrations required for AEA to stimulate TRPV1 are usually higher than those necessary for CB_1_ stimulation [29], and that other acylethanolamides that are substrates for FAAH are less potent than AEA at activating these channels [30]. On the other hand, the substrate concentration requirements for several enzymes to efficiently catalyze the oxygenation of AEA are higher than those required for this endocannabinoid/endovanilloid to be hydrolyzed by FAAH [15], [17], suggesting that such enzymes may play a role in AEA inactivation only when FAAH is fully inhibited and AEA levels increased beyond a certain threshold. As a consequence, the doses of URB597 used to inhibit FAAH, and the subsequent extent of elevation of AEA levels, become an important issue to investigate. We tested, here and previously, URB597 at two different concentrations, 10 µg and 100 µg, and observed that spinal AEA reduces neuropathic pain via CB_1_ or TRPV1, depending on its local concentration, bioavailability, tissue level elevation and subcellular distribution [23]. Furthermore, a previous study [21] showed that, depending on the dose used for site injections into the periaqueductal gray (PAG), or on the interval of time since its administration, URB597 either suppresses or increases thermal nociception in healthy rats *via* TRPV1 or CB_1_ receptors, respectively (please refer to [21],[31] for detailed mechanism description). In the present study, the anti-allodynic and anti-hyperalgesic actions of URB597 at the 200 µg dose were antagonized almost uniquely by I-RTX. It was, therefore, surprising to find that, following this treatment, the spinal levels of endogenous AEA were decreased, rather than being further increased. On the other hand, a significant augmentation of 2-AG, PEA and OEA concentrations was detected instead. The concomitant increase of 2-AG levels, an endocannabinoid FAAH substrate already reported to be up-regulated following local injections of URB597 (see [21] and [32] for review), would explain why CB_1_ antagonism with AM251 still tended to reduce at 15 and 60 min, and significantly attenuated at 30 min, URB597-mediated analgesia in the Hargreaves test, thus suggesting the presence of a transitory involvement of CB_1_ receptors in some of URB597 effects in this test. Indeed, the increase of brain and spinal cord 2-AG levels obtained by the administration of inhibitors of 2-AG inactivation by monoacylglycerol lipase was previously shown to reduce mechanical and cold allodynia in the CCI model [33], [34]. On the other hand, the increase by URB597 of the levels of OEA and PEA, which are also substrates for FAAH, would explain why the effects of the high dose of the FAAH inhibitor were antagonized by I-RTX. In fact, a role for OEA in inflammatory pain has been demonstrated [35], and it has been proposed that this compound acts not only *via* nuclear peroxisome proliferator-activated receptor (PPAR)-α and-γ, but also TRPV1 ([30], [36], [37] for review). Also PEA exhibits anti-allodynic and anti-hyperalgesic effects in a murine model of neuropathic pain *via* multiple mechanisms [38]. PEA can act as an allosteric enhancer of AEA actions at TRPV1 [39] or activate and desensitize this channel, in part directly and in part via PPAR-α activation [40]. Thermal hyperalgesia and mechanical allodynia in CCI mice were relieved by repeated administration of PEA [38], and for this reason this compound is thought to be involved in the endogenous protective mechanisms that are activated in the body as a result of different types of tissue damage and hence also in neuropathic pain [41].

To explain the decrease in AEA levels induced by the highest dose of URB597, we hypothesized that, following full inactivation of FAAH, alternative pathways of AEA metabolism might come into play to form AEA metabolites that inhibit nociception. Because of its arachidonate-containing chemical structure, AEA is not only subjected to hydrolysis but can also be metabolized by several of the same enzymes that are responsible for arachidonic acid oxidation, including COX-2 and lipoxygenases [42]. COX-2 is responsible for catalyzing the oxidation of AEA and 2-AG into various prostamides and prostaglandin-glycerol esters, respectively [43]. A major COX-2 metabolite of AEA, prostamide F_2α_, was recently identified in the spinal cord of mice with knee inflammation. However, this compound was found to contribute to analgesia rather than counteracting it, in a way mediated by specific receptors [24]. Previous data had shown that prostamide F_2α_ is inactive at TRPV1 channels [16], and therefore COX-2 inhibitors were not tested in the present study. Oxidative metabolism of AEA by 12-LOX and 15-LOX, instead, results in the formation of 12- and 15-hydroperoxy-AEA, respectively [17], [44], which exert their biological activities via established receptors, including the cannabinoid receptors, PPAR-α and TRPV1 [17], [18], [42], [45]. Indeed, the TRPV1-mediated contractile action of AEA on guinea-pig isolated bronchi was attenuated by LOX inhibitors [18]. Thus, AEA lipoxygenation, together with the increase in OEA and PEA levels, might also have contributed to the TRPV1-mediated effects of the high dose of URB597. Accordingly, we observed here that CCI induced the up-regulation of spinal 15-LOX mRNA, and thus we investigated the expression of this enzyme in the DH of spinal cord of CCI rats. Using western blot analyses, we found that spinal 15-LOX protein levels were significantly up-regulated. Furthermore, using immunofluorescence techniques, we observed a very high degree of co-localization among FAAH, TRPV1 and 15-LOX in more than 50% of the cell bodies in lamina II, which was instead nearly absent in sham rats. We suggest that the activity of 15-LOX may be controlled at two different molecular levels: 1) regulation of gene expression, which occurs on both sides of the DH; and 2) regulation of mRNA translation to the active protein, only on the injured side.

Based on these data, we hypothesize that, when FAAH is fully inhibited in certain spinal neurons, AEA levels can reach threshold levels to be recognized by 15-LOX in the same neurons and produce 15(*S*)-hydroxy-AEA, which is capable of acting at TRPV1 in the same cells, desensitize it and produce analgesic effects. Accordingly, when baicalein was co-administered with URB597, the analgesic effect was partly lost, which also suggests that, when both FAAH and LOX enzymes are inactivated, AEA can still be metabolized to yet other compounds that might: 1) either have no antinociceptive action and represent a new mechanism for the termination of AEA signaling [46]; or 2) even exacerbate pain, as in the case of prostamideF_2α_ [24]. In agreement with the hypothesis that 15-LOX metabolizes AEA to TRPV1 agonists, we report here that 15(*S*)-hydroxy-AEA is relatively potent and quite efficacious at stimulating intracellular calcium elevation in a TRPV1-mediated manner in HEK-293 cells overexpressing the rat recombinant TRPV1, but not in wild-type HEK-293 cells, and equally potent at desensitizing TRPV1 to the action of capsaicin. This finding is in agreement with a previous study indicating that 15(*S*)-hydroxy-AEA can displace the specific binding of [^3^H]-resiniferatoxin (a high affinity TRPV1 ligand) to mouse brain membranes [47]. Interestingly, in this previous study, the authors also reported that 15(*S*)-hydroxy-AEA stimulates the activity of an acylethanolamide-biosynthesizing enzyme, and inhibits FAAH as well as the activity of a 2-AG-biosynthesizing enzyme [47]. Thus, the formation of 15(*S*)-hydroxy-AEA might reinforce further and prolong the inhibition of FAAH, contribute to elevate the levels of TRPV1-active acylethanolamides by both inhibiting their degradation and stimulating their biosynthesis, sustain its own biosynthesis from AEA, and counteract CB_1_ activation by 2-AG, thereby leading, together with OEA and PEA, to an overall prolonged activation/desensitization of TRPV1 signaling, with subsequent anti-hyperalgesic and anti-allodynic effects in neuropathic rats. Notably, also 15(*S*)-HETE, a major arachidonic acid metabolite from the 15-lipoxygenase pathway, was shown to have anti-inflammatory roles [48]. Furthermore, varying expression patterns of the two major 15-LOX isoforms may underlie different production patterns of downstream lipid mediators (e.g., 15-HETE and 13-HODE) in different tissues [49], thus contributing in different, or even opposing ways, to pain signaling or its modulation.

In conclusion, we have provided here a new example of the complex network surrounding AEA signaling and of the potential complications of the pharmacological manipulation of the levels of this endocannabinoid/endovanilloid mediator, which may result in the readaptation of this network, whilst still leading, at least in neuropathic rats, to the counteraction of hyperalgesia and allodynia. Our findings support the use of high doses of intrathecal FAAH inhibitors as analgesic drugs for chronic pain.

## Summary

Following full inhibition of its hydrolysis, AEA might be metabolized to 15(*S*)-hydroxy-AEA, which, together with OEA and PEA, may produce TRPV1-mediated anti-hyperalgesic and anti-allodynic effects in neuropathic rats.

## Materials and Methods

### Animals

Male Wistar rats (Charles River, Hamburg, Germany), initially weighing between 250–275 g, were used for all experiments. Animals were housed individually, under a 12∶12 h light/dark cycle and free access to food and water. All experiments were carried out between 9∶00 am and 12∶0 am. All animals were allowed to acclimatize to their holding cages for 3–4 days before any behavioral, or surgical procedures were carried out. Experimental procedures followed the guidelines of the IASP [50] and were approved by the Local Bioethics Committee of the Institute of Pharmacology (Cracow, Poland). Care was taken to reduce both number of animals used and suffering during the experiments.

### Intrathecal Catheterization

Rats were chronically implanted with intrathecal (i.t.) catheters according to [51] under pentobarbital anesthesia (60 mg/kg; i.p.). The catheter (PE 10,Clay Adams) sterilized by immersion in 70% ethanol, and flushed with sterile water prior to insertion was carefully introduced through atlanto-occipital membrane to the subarachnoid space at the rostral level of the spinal cord lumbar enlargement (L4–L6). Rats exhibiting postoperative neurological deficits (approximately 5–10%) were excluded from the study.

### Sciatic Nerve Surgery

Peripheral neuropathy was induced by chronic constriction injury (CCI) to the sciatic nerve, as described by [52]. The sciatic nerve injury was performed under sodium pentobarbital anesthesia (60 mg/kg, i.p.)7 days after implantation of the i.t. catheter. The biceps femoris and the gluteus superficialis were separated, and the right sciatic nerve was exposed. Proximal to the sciatic trifurcation, about 7 mm of nerve was freed of adhering tissue and the injury was produced by tying four loose ligatures (4/0 silk, 1 mm spacing) around the sciatic nerve. The twitch of muscles during ligation prevented us from applying it too strongly. The total length of nerve affected was 4–5 mm. In sham-operated rats, the sciatic nerve was visualized and left intact.

### Nociceptive Testing

On the day of the experiment, behavioral testing was carried out over 30 min before, then over a 1 h period (15, 30 and 60 min) after i.t. drug administration. All experiments were conducted at day 7 after the sciatic nerve ligation in order to avoid the inflammatory component of pain, which could appear in initial state after injury.

#### Tactile allodynia (von Frey test)

For the assessment of tactile allodynia, rats were tested for their foot withdrawal threshold in response to automatic von Frey apparatus (Dynamic Plantar Aesthesiometer Cat. No.37400, Ugo Basile Italy). Rats were placed in plastic cages with wire net floor 5 min before the experiment. The von Frey filament was applied to the midplantar surface of the ipsilateral hind paw and the measurements of applied mechanical force were taken automatically. Hind paws were tested two times in 3-min intervals, and the response mean value was calculated. The strength of the von Frey stimuli in our experiments ranged from 0.5 to 26 g.

#### Thermal hyperalgesia (Hargreaves test)

For the assessment of paw withdrawal latency (PWD) to a noxious thermal stimulus the Analgesia Meter (mod 33, IITC INC., Landing, NJ) was used [53]. On the day of experiment, each animal was placed in a plastic cage with a heated glass floor. After 5 min of habituation, a noxious thermal stimulus - light beam was focused onto the plantar aspect of a hindpaw until the animal lifted a paw away from the heat source. The paw withdrawal latency was automatically displayed to the nearest 0.1 s. A cut-off latency of 20 s was used to avoid tissue damage. The latency of nociceptive reaction was measured in seconds under basal condition and after i.t. treatment with drugs.

#### Cold hyperalgesia

The hyperalgesia was assessed using the cold plate test (Cold/Hot Plate Analgesia Meter No.05044 Columbus Instruments, USA). The temperature of the cold plate was kept at 5°C, the cut-off latency was 30 s. The rats were placed on the cold plate and the time until lifting of the hind paw was recorded, the injured paw exhibiting a lower reaction latency.

### Drugs

Anandamide (AEA), AM251, 5'-Iodoresiniferatoxin (I-RTX) and baicalein were purchased from Tocris (Bristol, UK) and URB597 from Sigma-Aldrich (Poznan, Poland). 15(*S*)-hydroxy-AEA and capaicin were purchased from Cayman Chemical (Ann Arbor, MI, USA). URB597 and AM251 were made up in a vehicle solution comprising (v/v%) 18% dimethyl sulphoxide (DMSO), 1% ethanol, 1% Tween-80 and 80% saline. Stock solution of AEA and I-RTX were prepared in ethanol and furtherdiluted with saline. URB597 was used at the dose of 200 µg (58 mM); 1.5 µg of baicalein (555 µM), 5 µg of I-RTX (0.66 mM); 30 µg of AM251 (5.4 mM). Each dose was injected intrathecally in volume of 10 µl.

### Experimental Design

Groups of 8 animals per treatment were used, with each animal used for one treatment only. In behavioral experiments vehicle-treated (i.t.) CCI animals formed the control group. We did not observe changes in their response values throughout the testing period (0–60 min). Additionally, in all testing procedures we measured also the contralateral paw response for all tested drugs. These values were not altered by the treatment applied (data not shown).

The first series of experiments was performed to characterize the effect of pharmacological elevation of endogenous spinal AEA levels and, to examine whether TRPV1 or CB_1_ receptor antagonists would block the behavioral effects caused by the FAAH inhibitor. To this end, rats were injected with URB597 alone, or i.t. administration of TRPV1 or CB_1_ antagonists (I-RTX or AM251) was followed by URB597 15 min later.

In the second series of experiments we assessed the possible influence of lipoxygenation products on the behavioral effects elicited by URB597 and again assessed the involvement of TRPV1 or cannabinoid CB_1_ receptors in the observed effects, by blocking these receptors with theirs specific antagonists.

All behavioral paradigms included time-course testing, ranging from 0 to 60 min after i.t. drug administration. For the clarity of graphs, most representative data relate to 30 min after i.t. administration.

The doses of I-RTX and AM251 tested for antagonism of AEA and/or URB597 effects were selected in order not to have any effect on allodynia and hyperalgesia *per se* (for details see [23]).

### Tissue Collection, RNA Preparation and Quantitative Real-time PCR

Seven days after CCI rats were decapitated,spinal cords were dissected rapidly. The samples were placed in individual tubes with the tissue storage reagent RNA later (Qiagen Inc., Valencia, CA, USA), frozen on dry ice, and stored at −70°C until RNA isolation. Samples were thawed at room temperature and homogenized in 1 ml Trizol reagent (Invitrogen, Carlsbad, CA, USA). RNA isolation was performed according to the manufacturer's protocol. The total RNA concentration was measured using a NanoDrop ND-1000 Spectrometer (NanoDrop Technologies Inc., Montchanin, DE, USA). RNA quality was determined by chip-based capillary electrophoresis using an RNA 6000 Nano LabChip Kit and Agilent Bioanalyzer 2100 (Agilent, Palo Alto, CA, USA). Total RNA from ipsilateral lumbar (L4–L6) dorsal part of the spinal cord of two animals was pooled.

Reverse transcription was performed with Omniscript Reverse Transcriptase enzyme (Qiagen) at 37°C for 60 minutes. The reaction was carried out in the presence of the RNase inhibitor rRNAsin (Promega, Madison, WI, USA), and an oligo(dT_16_) primer (Qiagen) was used to selectively amplify mRNA. qPCR reactions were performed using Assay-On-Demand TaqMan (Applied Biosystems, Foster City, CA, USA). The following assays were used Rn00563172_m1 (*Alox5*), Rn01461082_m1 (*Alox12*), Rn00696151_m1 (*Alox15*) and Rn01527838_m1 (*Hprt*). Reactions were run on a Real-Time PCR iCycler device (BioRad, Hercules, CA, USA) with the 3.0 a software version. Threshold cycle values were calculated automatically with default parameters. The amplification efficiency for each assay was determined by running a standard dilution curve. Expression of the *Hprt1* transcript with stable level between the control and CCI groups was quantified to control for variation in cDNA amounts. The threshold cycle (CT) value for each gene was normalized with the CT value of *Hprt*. The abundance of RNA was calculated as 2^-(normalized threshold cycle)^.

### Western Blot

The lumbar (L5–L6) region of the spinal cord was removed and divided into ipsilateral and contralateral parts with respect to the side of injury or left and right in the case of naive mice. Tissue samples were homogenized in RIPA buffer with proteases and phospatases inhibitors (Sigma-Aldrich) and cleared by centrifugation (10,000 ×g, 4°C, 30 min). The protein concentration in the supernatant was determined using the BCA Protein Assay Kit (Sigma-Aldrich). Samples containing 30 µg of protein were heated for 8 min at 99°C in loading buffer (50 mMTris–HCl, 2% SDS, 2% β-mercaptoethanol, 8% glycerol and 0.1% bromophenol blue) and resolved by SDS-PAGE on 10% polyacrylamide gels. After gel electrophoresis, proteins were electrophoretically transferred to PVDF membranes (Trans-Blot; Bio-Rad). The blots were blocked using 5% blocking buffer (2.5% albumin +2.5% non-fat drymilk)in TBST (Tris-buffered saline with 0.1% Tween 20) for 1 h. Blots were incubated with rabbit polyclonal anti-LOX15 at 0,5 ug/ml concentration (Aviva Systems Biology Corp.). Blots were incubated overnight at 4°C with primary antibodies and then incubated with a peroxidase-conjugated secondary antibody (goat anti-rabbit/mouse IgG, Bio-Rad) at a dilution of 1∶1000 for 1 h at room temperature. After three 10 min washes in TBST, immunocomplexes were detected using a Immuno-Star HPR kit (Bio-Rad) and visualized using a Fujifilm LAS-1000 fluorimager system. The blots were stripped and reprobed with a mouse polyclonal anti-GAPDH antibody (1∶1000 dilution) (Millipore) as loading control. Relative levels of immunoreactivity were quantified using the Fujifilm Image Gauge software.

### Measurement of Anandamide and Other Spinal Fatty Acid Amides by *liquid chromatography*–mass Spectrometry (LC-MS)

A sham operation of the sciatic nerve (nerve was exposed, but not touched) formed a control group for all LC-MS experiments. Sham animals did not produce neuropathic symptoms, i.e. no changes in withdrawal repose to mechanical and thermal stimulation were observed.Seven days after the induction of the CCI ipsilateral and contralateral lumbar sections of the spinal cord (n = 6) were sectioned and homogenized in 5 vol of chloroform:methanol:Tris-HCl 50 mM (2∶1∶1) containing 10 pmol of d8-AEA, d4-palmitoylethanolamide (PEA), d5–2-arachidonoylglycerol (2-AG), d4palmitoylethanolamide (PEA) and d4–oleoylethanolamide (OEA). Homogenates were centrifuged at 13000×g for 16 min (4°C), the aqueous phase plus debris were collected and extracted again twice with 1 vol of chloroform. The organic phases from the three extractions were pooled and the organic solvents evaporated in a rotating evaporator.

Lyophilized extracts were resuspended in chloroform:methanol (99∶1, v v–1). The solutions were then purified by open bed chromatography on silica as previously described [54]. Fractions eluted with chloroform:methanol (9∶1, v v−1) and containing AEA, PEA, 2-AG and OEA, were collected and the excess solvent evaporated with a rotating evaporator, and aliquots analysed by isotope dilution-liquid chromatography/atmospheric pressure chemical ionization/mass spectrometry (MS) carried out under conditions described previously [54] and allowing the separations of 2-AG, PEA, AEA and OEA. Results are expressed as nmol per g of wet tissue, or pmol per mg in the case of 2-AG.

### Immunofluorescence Microscopy

Animals (n = 3 CCI and n = 3 sham operated rats) were deeply anaesthetized with pentobarbital and perfused transcardially with saline followed by ice-cold 4% paraformaldehyde in 0.1 M phosphate buffer (PB), pH 7.4. Spinal cords were removed, post-fixed for 2 h and then washed. Tissues to be cut at cryostat were cryoprotected overnight in PB containing 30% (w/v) sucrose at 4°C. Serial cryostat sections were cut at 14 mm and mounted onto gelatine-coated slides (Mezel, Germany). For double immunofluorescence, serial sections were incubated for 1 h in 10% normal donkey serum (NDS, Jackson Immunoresearch Laboratories, West Grove, PA) in PB containing 0.3% Triton X-100 (block solution). Subsequently the sections were incubated for 2 days at 4°C in a humid chamber with the respective polyclonal antibodies (all diluted in block solution). All sections were processed for guinea pig anti-TRPV1 receptor immunostaining (1∶400, Novus Biologicals, Cambridge, UK) as well as goat anti-FAAH (1∶200, Santa Cruz, Heidelberg, Germany) and rabbit anti-15-LOX (1∶200, Abcam, Cambridge, UK) immunostaining. After three washes in PB, triple immunofluorescence was revealed by incubation for 2 h in the appropriate fluorochrome-conjugated secondary antibody: Alexa Fluor488 donkey anti-guinea pig, Alexa Fluor546 donkey anti-goat and Alexa Fluor350 donkey anti-rabbit all diluted 1∶200 in NDS. Thereafter, sections were washed with PB and coverslipped with Aquatex mounting medium (Merck, Darmstadt, Germany).

Controls of immunostaining included: (1) preabsorption of diluted antibodies with their respective immunizing peptides, and (2) omission of either the primary antisera or the secondary antibodies. These control experiments did not show notable staining. The sections processed for immunofluorescence were studied with an epifluorescence microscope (Leica DMI6000). Images were acquired using the digital camera Leica DFC 320 connected to the microscope and the image analysis software Leica IM500, which allows both single and merged pictures acquisitions. Digital images were processed in Adobe Photoshop, with brightness and contrast being the only adjustments made.

Quantification of immunoreactivity.Quantification of the mean percentage value of the number of neurons TRPV1/FAAH, TRPV1/15-LOX, 15-LOX/FAAH and TRPV1/15-LOX/FAAH labeled was performed, by an observer blinded to the experimental protocols. The total number of neurons was counted bycresyl violet in the lamina II/III of DH of adjacent immunolabelled sections and only in the cells whose nucleus, unstained or lightly stained, was in the focal plane. The level of section evaluated for immunohistochemistry study covered, bilaterally, the entire extension of lumbar DH on a total of 20 sections per animal (3 animals per group).

### Intracellular Calcium Assays

HEK-293 (human embryonic kidney) wild type and stably transfected cells overexpressing the rat TRPV1-cDNA cells were grown as monolayers in EMEM supplemented with nonessential amino acids, 10% fetal bovine serum, and 2 mM glutamine, maintained under 5% CO2 at 37°C plated on 100-mm diameter Petri dishes. On the day of the experiment, the cells were loaded for 1 h at 25°C with the methyl ester Fluo-4-AM (Invitrogen), 4 µM in DMSO containing 0.02% Pluronic F-127 (Invitrogen). After loading, cells were washed twice in Tyrode’s buffer (145 mMNaCl, 2.5 mMKCl, 1.5 mM CaCl_2_, 1.2 mM MgCl_2_, 10 mM D-glucose, and 10 mM HEPES, pH 7.4), resuspended in the same buffer, and transferred to the quartz cuvette of the spectrofluorimeter (Perkin-Elmer LS50B) under continuous stirring. [Ca^2+^]_i_ was determined before and after the addition of various concentrations of test compound by measuring cell fluorescence (λ_EX_ = 488 nm, λ_EM_ = 516 nm). Curve fitting (sigmoidal dose-response variable slope) and parameter estimation were performed with GraphPad Prism® (GraphPad Software Inc., San Diego, CA). Potency was expressed as the concentration of test substance exerting a half-maximal agonist effect (i.e. half-maximal increases in [Ca^2+^]_i_) (EC_50_). The efficacy of TRPV1 agonists was first determined by normalizing their effect to the maximum Ca^2+^ influx effect on [Ca^2+^]_i_ observed with application of 4 µM ionomycin. Desensitizing behaviour was evaluated against capsaicin (0.1 µM) by adding the test compound in the quartz cuvette 5 min before stimulation of cells with agonists. Data are expressed as the concentration exerting a half-maximal inhibition of agonist-induced [Ca^2+^]_i_ elevation (IC_50_), which was calculated again using GraphPad Prism® software. The effect on [Ca^2+^]_i_ exerted by agonist alone was taken as 100%. Dose response curves were fitted by a sigmoidal regression with variable slope. All determinations were performed at least in triplicate.

### Statistical Analyses

All data are reported as mean ± SEM and were analyzed using one-way analysis of variance (ANOVA) in the case of behavioral experiments and two-way analysis of variance in the case of qPCR, western blot and LC-MS analyses, with follow-up comparisons using Bonferroni post-hoc test. Results with *p*<0.05 were considered significant.
